# Dietary fiber intake and non-alcoholic fatty liver disease: The mediating role of obesity

**DOI:** 10.3389/fpubh.2022.1038435

**Published:** 2023-01-06

**Authors:** Yu Zhu, Hu Yang, Yaozong Zhang, Songxian Rao, Yufeng Mo, Honghua Zhang, Shaoxian Liang, Zhuang Zhang, Wanshui Yang

**Affiliations:** ^1^Department of Nutrition, School of Public Health, Anhui Medical University, Hefei, Anhui, China; ^2^School of Public Health, Wannan Medical College, Wuhu, Anhui, China

**Keywords:** dietary fiber, non-alcoholic fatty liver disease, liver function parameters, mediation analysis, cross-sectional study

## Abstract

**Background and aims:**

Dietary pattern rich in fiber is negatively associated with the risk of non-alcoholic fatty liver disease (NAFLD). Meanwhile, obesity is a known predisposing factor for NAFLD. Nutrient-focused research can enhance the mechanistic understanding of dietary effects. We thus hypothesized that higher dietary fiber intake was associated with lower risk of NAFLD through the mediating role of obesity.

**Methods:**

In this nationwide cross-sectional study, dietary fiber was surveyed using two 24-h recalls. NAFLD and clinically significant fibrosis (CSF) were determined by vibration-controlled transient elastography. Multivariable logistic and linear regression were applied to investigate the association of dietary fiber with NAFLD, CSF, and liver function parameters. We used counterfactual-based mediation analysis to estimate the direct and indirect effect of dietary fiber on NAFLD.

**Results:**

Of the 3,974 participants, ~36.86% and 7.78% of participants were diagnosed with NAFLD and CSF. Compared with participants among the lowest tertile, the highest tertile of dietary fiber consumption was associated with lower odds of NAFLD (OR = 0.81; 95% CI: 0.66–0.98; *P*_*overall*_ = 0.019). Dietary fiber intake appeared to be linked with lower odds of CSF (OR_*Tertile*3*vs*.*Tertile*1_ = 0.81; 95% CI: 0.58–1.14; *P*_*overall*_ = 0.107). Mediation analysis showed that obesity fully mediated the association of dietary fiber with NAFLD. Dietary fiber was associated with improved hepatic parameters.

**Conclusions:**

The findings indicated that increasing dietary fiber intake could confer a greater benefit to protect against NAFLD. Translating these findings regarding dietary fiber into dietary advice might be an attractive strategy for NAFLD prevention.

## Introduction

Non-alcoholic fatty liver diseases (NAFLD), characterized by a certain degree of steatosis arising in the absence of excessive alcohol consumption and other known causes of liver disease (1), approximately affects a third of the world population (2). Growing evidence indicates that NAFLD is the hepatic manifestation of metabolic syndrome (MetS) (3). Paralleling the global epidemic of obesity and diabetes, the prevalence of NAFLD is growing dramatically over the past three decades in the US (4), and is projected to increase further. Although there are various existing drugs that have been considered in the management of NAFLD, lifestyle management such as healthy diet remains first-line treatments for NAFLD (5). Diet is the main driver of triglycerides accumulation in hepatocytes (6). Identifying dietary factors that reduce the risk of NAFLD is of importance. However, to date, such research is limited.

A previous study suggested a protective role for higher adherence to plant-based diet (PDI) against NAFLD (6). Furthermore, the Mediterranean diet has been shown to prevent NAFLD (7), and alleviate hepatic steatosis as well as fibrosis in the regression of NAFLD (8). On the other hand, several epidemiological studies demonstrated that high red meat and processed meat consumption might increase the risk of NAFLD (9, 10). PDI and Mediterranean diets are hallmarked by a fiber-rich diet. Consumption of dietary fiber has been associated with lower risk of obesity (11, 12), which was a known predisposing factor for NAFLD. A meta-analysis synthesizing 21 cohort studies with a total of 381,655 participants suggested that obesity independently led to a 3.5-fold increased risk of developing NAFLD compared with normal weight (13). Effect estimate on association between dietary factor and NAFLD appeared to be largely attenuated when obesity was adjusted (7, 14), suggesting that obesity might be on the causal pathway of the relation (a mediator) (15). Because of complex interplay between diet, nutrient-focused research can enhance the mechanistic understanding of dietary effects (16). Taken together, we hypothesized that intake of dietary fiber lowered the risk of NAFLD, and obesity might play a mediating role in linking dietary fiber to NAFLD. To our knowledge, three studies have reported inverse associations between dietary fiber intake and NAFLD (17–19). One with a nested case-control study identified NAFLD through linkage to the Medicare claims (17), while NAFLD was determined using liver ultrasonography and fatty liver index (FLI) in the other two cross-sectional studies (18, 19). However, there has been no epidemiological study to evaluate the association of dietary fiber intake with NAFLD and clinically significant fibrosis (CSF) determined by vibration-controlled transient elastography (VCTE), one of the most accurate methods to detect hepatic steatosis and fibrosis (20). Meanwhile, given that the association was mediated by obesity, how dietary fiber exerted its effect *via* direct and indirect pathways have not been evaluated.

To add more evidence, we investigated the association of dietary fiber intake with NALFD and CSF determined by VCTE in a nationally representative sample of US adults. Furthermore, mediation analysis was applied to assess the extent to which the effect of dietary fiber acted on NAFLD through obesity.

## Methods

### Study population

In this study, participants were selected from the 2017 to 2018 cycle of US National Health and Nutrition Examination Survey (NHANES), which was a cross-sectional survey conducted by the National Center for Health Statistics (NCHS) of the Centers for Diseases Control and Prevention (CDC) in the United States. More details on the survey protocol of NHANES have been described elsewhere (21). The study protocol was approved by NCHS Research Ethics Review Board (IRB: Protocol #2011-17; Protocol #2018-01), and the written informed consent was obtained from all participants.

A total of 9,254 participants were enrolled in the survey. Individuals aged ≥18 years old were included in this study. Individuals were excluded if they (i) had a missing dietary data (*n* = 873) or an unreliable energy intake (22) (defined as <600 or >3,500 kcal/day for women; <800 or >4,200 kcal/day for men, *n* = 221); (ii) had hepatitis B virus infection (the presence of HBsAg, *n* = 20) or hepatitis C virus infection (both hepatitis C antibody and RNA being positive*, n* = 82); (iii) had significant alcohol consumption (>3 drinks/d for men and >2 drinks/d for women*, n* = 116), (iv) underwent VCTE detection with unreliable results (*n* = 315) or did not receive VCTE detection (*n* = 255, Supplementary Figure 1).

### Dietary assessment

Dietary intake was quantified *via* two 24-h dietary recalls. A first 24-h dietary recall was performed in-person in the NHANES Mobile Examination Center (MEC), and the second 24-h dietary recall was conducted by telephone 3–10 days after the first recall. Food energy and nutrients were calculated based on the US Department of Agriculture (USDA), Food and Nutrient Database for Dietary Studies 2017–2018 (FNDDS 2017–2018). Total dietary fiber was calculated by multiplying the weight of each food consumed by the nutrient content of that food and summing it across foods. The intake of dietary fiber was averaged when participants had twice dietary recalls. We adjusted dietary fiber intake for total energy intake using the nutrient density method (intake per 1,000 kcal) to decrease measurement errors and represent the dietary composition.

### Definition of mediator

In this study, obesity was hypothesized to be a mediator of the association between dietary fiber and NAFLD. Anthropometric measurement (height and weight) was conducted in MEC. Body mass index (BMI) was calculated as weight (kg) divided by the square of the height (m), and obesity was defined as BMI ≥25.0 (kg/m^2^).

### Additional covariates

Standardized questionnaires were administrated through household interviews to collect demographic characteristics including age, sex, race/ethnicity, educational level, smoking, physical activity, and income. Physical activity was calculated by the sum of activities every week and was expressed in metabolic equivalent tasks (METS)-hours/week. Family income was defined as the ratio of family income to poverty. Additionally, hypertension was defined if individuals (i) reported a history of hypertension; or (ii) had a systolic blood pressure (SBP) ≥140 mmHg; or (iii) had a diastolic blood pressure (DBP) ≥ 90 mmHg. Diabetes was defined if individuals (i) reported a diagnosis of diabetes; or (ii) had a glycohemoglobin A1c (HbA1c) level ≥6.5%; or (iii) had a fasting glucose level ≥ 126 mg/dl; or (iv) had a random glucose level ≥ 200 mg/dl.

### Assessment of NAFLD and CSF

Vibration-controlled transient elastography (VCTE) was conducted by trained and certified technicians in MEC, using the FibroScan^®^ model 502 V2 Touch equipped with a medium or extra-large probe. Hepatic steatosis and fibrosis was assessed by controlled attenuation parameter (CAP) and liver stiffness measurement (LSM).

Non-alcoholic fatty liver diseases (NAFLD) was defined as a CAP score ≥285 dB/m in the absence of viral hepatitis and excessive alcohol intake (non-NAFLD vs. NAFLD), and a LSM score ≥8.6 kPa was used to define CSF (LSM ≥ 8.6 kPa) vs. non-CSF (LSM < 8.6 kPa) (23, 24).

### Laboratory assays and liver function parameters

After drawing and centrifuging the blood samples, the serum was aliquoted and stored at −70°C. Liver function parameters, including serum albumin, globulin, total protein, total bilirubin, alanine aminotransferase (ALT), aspartate aminotransferase (AST), gamma glutamyl transaminase (GGT), were also obtained from participants. All laboratory procedures were shown in detail elsewhere (25).

### Statistical analysis

Continuous variables were presented as mean ± standard deviation (SD) for normal distribution or median (*P*_25_, *P*_75_) for skewed distribution, and categorical variables were presented as percentages. We summarized characteristics of participants by NAFLD phenotype (non-NAFLD vs. NAFLD) with using *t*-test for continuous variables or chi-square test for categorical variables or Wilcoxon rank-sum test for ordinal variables. Several variables had few missing values, we used the approach of deleting rows to handle missing values (listwise deletion) due to a large sample size.

Dietary fiber intake and total energy were categorized into tertiles, we used multivariable logistic regression to evaluate the odds ratios (ORs) and 95% confidence intervals (CIs) for the association of dietary fiber with NAFLD and CSF. Model 1 did not adjust for the covariates, and Model 2 was performed with adjustment for age (18–39, 40–59, and ≥60), sex (male and female), smoking (never smokers and ever smokers), race/ethnicity (non-Hispanic White, non-Hispanic Black, and other races), education (less than high school, high school diploma, and more than high school), ratio of family income to poverty (<1.30, 1.30–3.49, and ≥3.50), physical activity (low level, moderate level, and high level), total energy (Tertile 1, Tertile 2, and Tertile 3), hypertension (yes and no), and diabetes (yes and no). We also applied restricted cubic splines with three knots to depict the dose-response curve between dietary fiber and NAFLD as well as CSF. Furthermore, considering departures from the normal distribution, all liver function parameters were natural logarithm transformed. Multivariable linear regression was performed to estimate the percentage change and 95% CIs for the associations of dietary fiber intake with liver function parameters.

We assumed that obesity was a mediator among the associations of dietary fiber intake with NAFLD phenotype. Because mediator and outcome were dichotomous events, a counterfactual-based mediation analysis was performed. We derived ORs of natural direct effect and natural indirect effect, and total effect was estimated as the product of the natural direct and indirect effect. Pathway diagram was shown in Supplementary Figure 2.

A two-sided *P* < 0.05 was considered to indicate statistical significance. All statistical analyses were performed using R software (version 4.1.0) and Mplus software (version 8.3).

## Results

### Characteristics of participants

A total of 3,974 participants aged from 18 to 80 years (mean age: 49.34 years; SD: 18.40 years) were included in this study, with 1,903 (47.89%) men and 2,071 (52.11%) women. The prevalence of NAFLD and CSF was 36.86% (1,465) and 7.78% (309). Compared with those who were free of NAFLD, participants with NAFLD were more likely to be older, male, less physically active as well as Hispanic or other races, and tended to consume more energy, have a history of smoking, hypertension, diabetes, and obesity. In addition, we observed that CAP, LSM, globulin, ALT, AST, and GGT were significantly higher in NAFLD participants than those with non-NAFLD, whereas a slight reduction in albumin was observed among NAFLD participants. More details were shown in Table 1.

**Table 1:** The characteristics of participants according to NAFLD phenotypes^a^.

**Characteristics**	**Overall^b^**	**NAFLD phenotypes**		***P***
		**Non-NAFLD**	**NAFLD**	
**No. of participants**	3,974	2,509	1,465	
**Age (%)**				<0.001
18–39	1,357 (34.15)	1,015 (40.45)	342 (23.34)	
40–59	1,190 (29.94)	669 (26.66)	521 (35.56)	
≥60	1,427 (35.91)	825 (32.88)	602 (41.09)	
**Male (%)**	1,903 (47.89)	1,098 (43.76)	805 (54.95)	<0.001
**Smoking (%)**				0.006
Never	2,429 (61.12)	1,590 (63.37)	839 (57.27)	
Former	936 (23.55)	519 (20.69)	417 (28.46)	
Current	609 (15.32)	400 (15.94)	209 (14.27)	
**Race (%)**				<0.001
Non-Hispanic white	1,385 (34.85)	848 (33.80)	537 (36.66)	
Non-Hispanic black	897 (22.57)	635 (25.31)	262 (17.88)	
Hispanic or other	1,692 (42.58)	1,026 (40.89)	666 (45.46)	
**Education (%)**				0.239
Less than high school	719 (18.12)	445 (17.76)	274 (18.75)	
High school diploma	979 (24.68)	610 (24.34)	369 (25.26)	
More than high school	2,269 (57.20)	1,451 (57.90)	818 (55.99)	
**Physical activity (%)**				<0.001
Low level	1,387 (35.18)	815 (32.77)	572 (39.29)	
Moderate level	379 (9.61)	240 (9.65)	139 (9.55)	
High level	2,177 (55.21)	1,432 (57.58)	745 (51.17)	
**Family income to poverty ratio (%)**				0.699
0–1.29	977 (27.82)	631 (28.50)	346 (26.66)	
1.30–3.49	1,423 (40.52)	876 (39.57)	547 (42.14)	
≥3.50	1,112 (31.66)	707 (31.93)	405 (31.20)	
Hypertension (%)	1,632 (41.78)	847 (34.38)	785 (54.44)	<0.001
Diabetes (%)	751 (18.90)	281 (11.20)	470 (32.08)	<0.001
Obesity (%)	1,595 (40.14)	649 (25.87)	946 (64.57)	<0.001
Total energy (kcal)	1,873.75 (1,443.12, 2,426.88)	1,845.50 (1,421.00, 2,402.50)	1,941.00 (1,483.50, 2,457.00)	0.014
Dietary fiber (g/1,000 kcal)	7.70 (5.73, 10.70)	7.72 (5.70, 10.70)	7.66 (5.82, 10.69)	0.862
CAP (dB/m)	262.00 (217.00, 307.00)	229.00 (199.00, 257.00)	323.00 (302.00, 352.00)	<0.001
LSM (kPa)	4.90 (4.00, 6.10)	4.60 (3.80, 5.60)	5.70 (4.50, 7.00)	<0.001
Albumin (g/dl)	4.10 (3.90, 4.30)	4.10 (3.90, 4.30)	4.10 (3.80, 4.30)	0.001
Globulin (g/dl)	3.10 (2.80, 3.30)	3.00 (2.80, 3.30)	3.10 (2.80, 3.40)	0.001
Total protein (g/dl)	7.20 (6.90, 7.40)	7.20 (6.90, 7.40)	7.10 (6.90, 7.40)	0.939
Total bilirubin (g/dl)	0.40 (0.30, 0.60)	0.40 (0.30, 0.60)	0.40 (0.30, 0.50)	0.180
ALT (IU/L)	17.00 (13.00, 25.00)	16.00 (12.00, 21.00)	22.00 (16.00, 32.00)	<0.001
AST (IU/L)	19.00 (16.00, 23.00)	18.00 (16.00, 22.00)	20.00 (16.00, 26.00)	<0.001
GGT (IU/L)	21.00 (14.00, 31.00)	18.00 (13.00, 26.00)	26.00 (18.00, 41.00)	<0.001

ALT, Alanine aminotransferase; AST, Aspartate aminotransferase; CAP, Controlled attenuation parameter; GGT, Gamma-glutamyl transaminase; LSM, Liver stiffness measurement; NAFLD, Non-alcoholic fatty liver disease.

a Values were presented as median(P_25_, P_75_) or percentages.

b The summing number for some categories are not 3,974 due to missing values.

### The associations of dietary fiber with NAFLD and CSF

**Figure 1:**
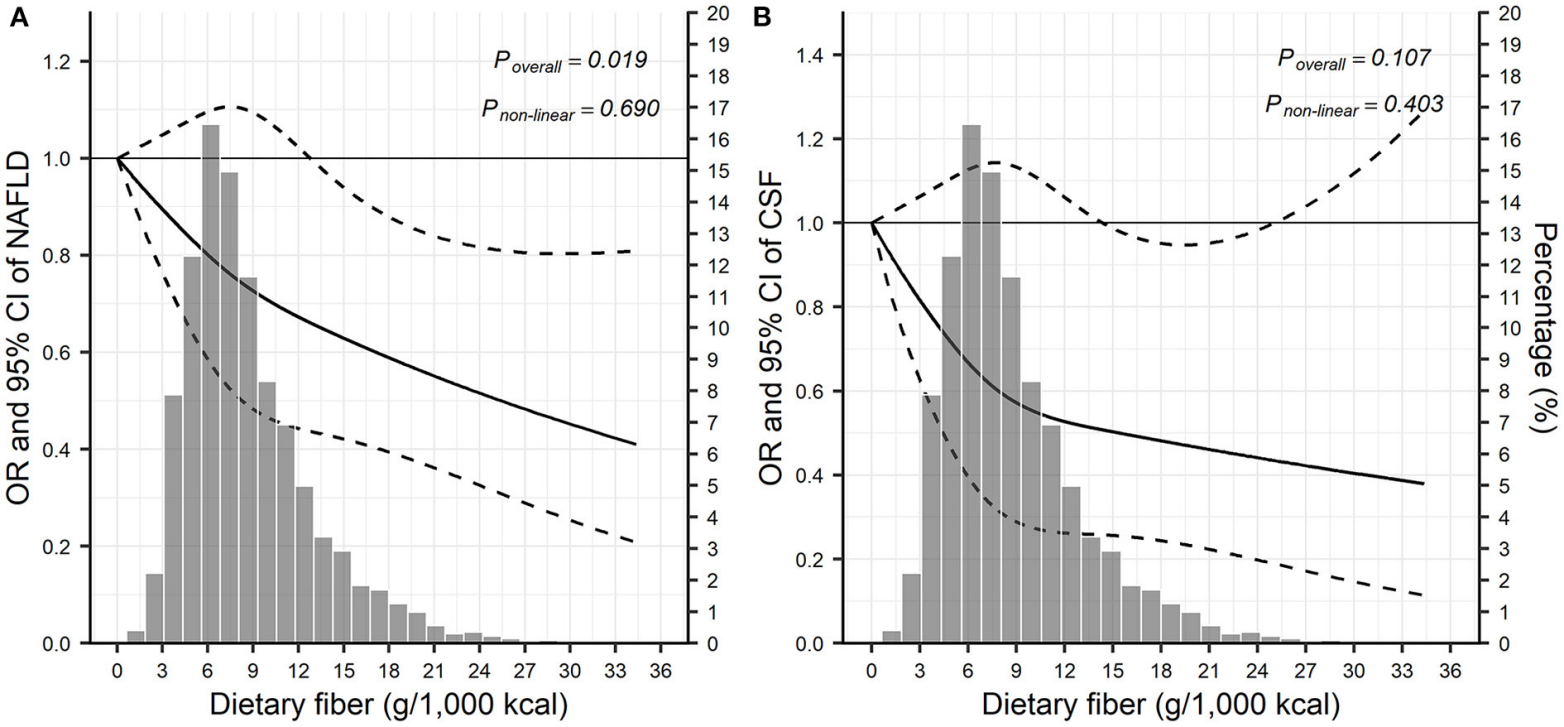
Visualization of the dose-response relationship between dietary fiber and liver disease based on restricted cubic splines^a^. **(A)** The relationship between dietary fiber and NAFLD; **(B)** the relationship between dietary fiber and CSF. CI, confidence interval; CSF, clinically significant fibrosis; NAFLD, non-alcoholic fatty liver disease; OR, odds ratio. ^a^Model was adjusted for adjusted for age, sex, smoking, race/ethnicity, education, ratio of family income to poverty, physical activity, total energy, hypertension, and diabetes.

In multivariable adjusted analyses, compared with the lowest tertile, the highest tertile of dietary fiber consumption was significantly associated with lower odds of NAFLD (OR = 0.81; 95% CI: 0.66–0.98; *P*_*trend*_ = 0.008; Table 2). As shown in the restricted cubic splines analysis (Figure 1A), the odds of NAFLD seemed to decline with the increase of dietary fiber consumption (*P*_*overall*_ = 0.019); non-linear trend, however, was not observed (*P*_*non*−*linear*_ = 0.690). Dietary fiber intake appeared to be linked with lower odds of CSF, however, this trend was only borderline statistical significance (OR_*Tertile*3*vs*.*Tertile*1_ = 0.81; 95% CI: 0.58–1.14; *P*_*overall*_ = 0.107 based on restricted cubic splines; Table 2, Figure 1B).

**Table 2:** The associations of dietary fiber consumption with NAFLD and CSF.

**Liver diseases**	**Dietary fiber consumption**			**Per 1-SD**	***P_*trend*_^c^***
	**Tertile 1**	**Tertile 2**	**Tertile 3**		
**NAFLD (ORs and 95% CIs)**					
Model 1^a^	1	1.07 (0.91–1.25)	1.01 (0.86–1.19)	0.98 (0.92–1.04)	0.458
Model 2^b^	1	0.95 (0.79–1.14)	0.81 (0.66–0.98)	0.89 (0.82–0.97)	0.008
**CSF (ORs and 95% CIs)**					
Model 1^a^	1	1.09 (0.82–1.44)	0.95 (0.71–1.26)	0.91 (0.80–1.03)	0.131
Model 2^b^	1	0.91 (0.67–1.25)	0.81 (0.58–1.14)	0.87 (0.75–1.01)	0.068

CIs, confidence intervals; CSF, clinically significant fibrosis; ORs, odds ratios.

a Model 1 did not adjust for the covariates.

b Model 2 was adjusted for age, sex, smoking, race/ethnicity, education, ratio of family income to poverty, physical activity, total energy, hypertension, and diabetes.

c The trend test was performed by using z-score in the models.

### Mediation effect of obesity on the association of dietary fiber with NAFLD

**Figure 2:**
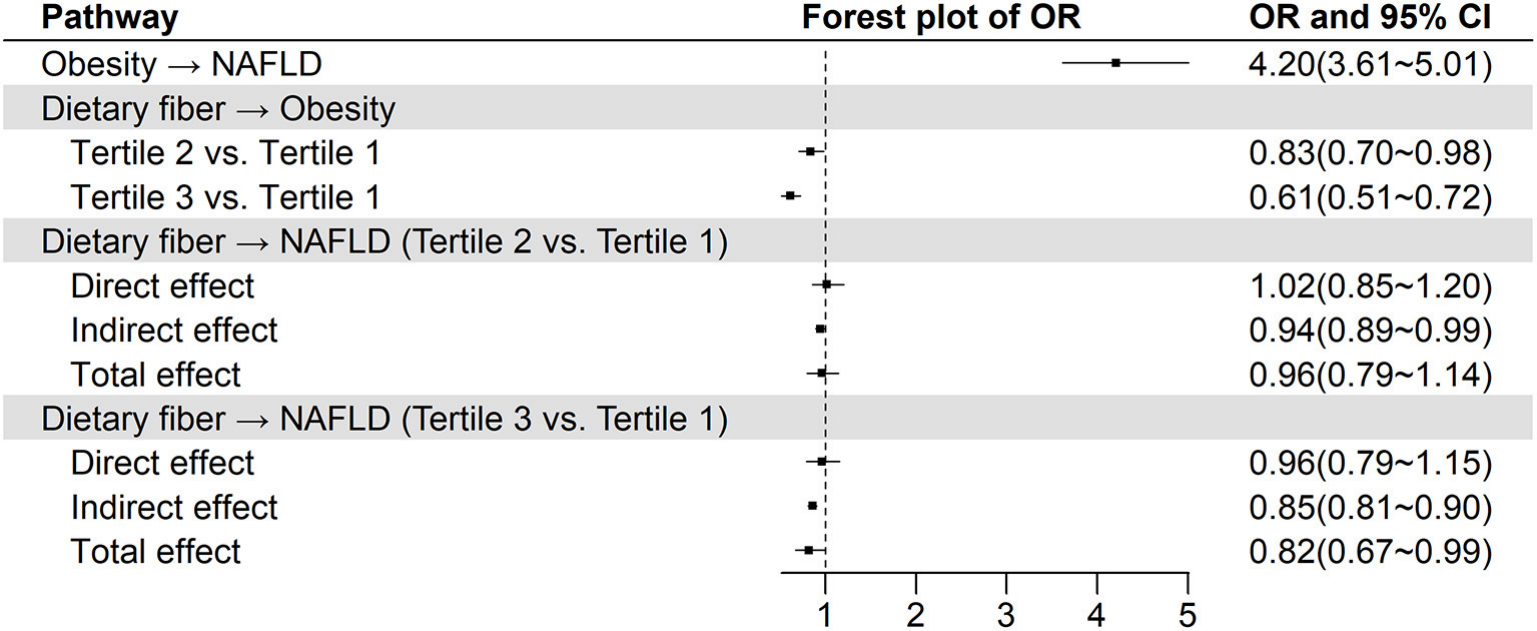
The relationship between dietary fiber and NAFLD from mediation analysis with controlling obesity as a mediator^a^. CI, confidence interval; NAFLD, non-alcoholic fatty liver disease; OR: odds ratio. ^a^Model was adjusted for adjusted for age, sex, smoking, race/ethnicity, education, ratio of family income to poverty, physical activity, total energy, hypertension, and diabetes.

The results of mediation analysis were presented in Figure 2. Obesity was associated with increased odds of NAFLD (OR = 4.20, 95% CI: 3.61–5.01). Compared with those with the lowest tertile intake of dietary fiber, individuals within medium tertile (OR = 0.83, 95% CI: 0.70–0.98) and the highest tertile (OR = 0.61, 95% CI: 0.51–0.72) had lower odds of obesity. Note that the direct effect of dietary fiber on NAFLD was not statistically significant. However, there were significant and protective indirect effect of dietary fiber (OR = 0.94, 95% CI: 0.89–0.99 for tertile 2 vs. tertile 1; OR = 0.85, 95% CI: 0.81–0.90 for tertile 3 vs. tertile 1) against NAFLD by affecting obesity. Taken together, the total effect of dietary fiber on NAFLD was statistically significant (OR = 0.82, 95% CI: 0.67–0.99) for tertile 3 vs. tertile 1. Because of non-significant association of dietary fiber with CSF, mediation analysis was not performed.

### The associations of dietary fiber with liver function parameters

**Figure 3:**
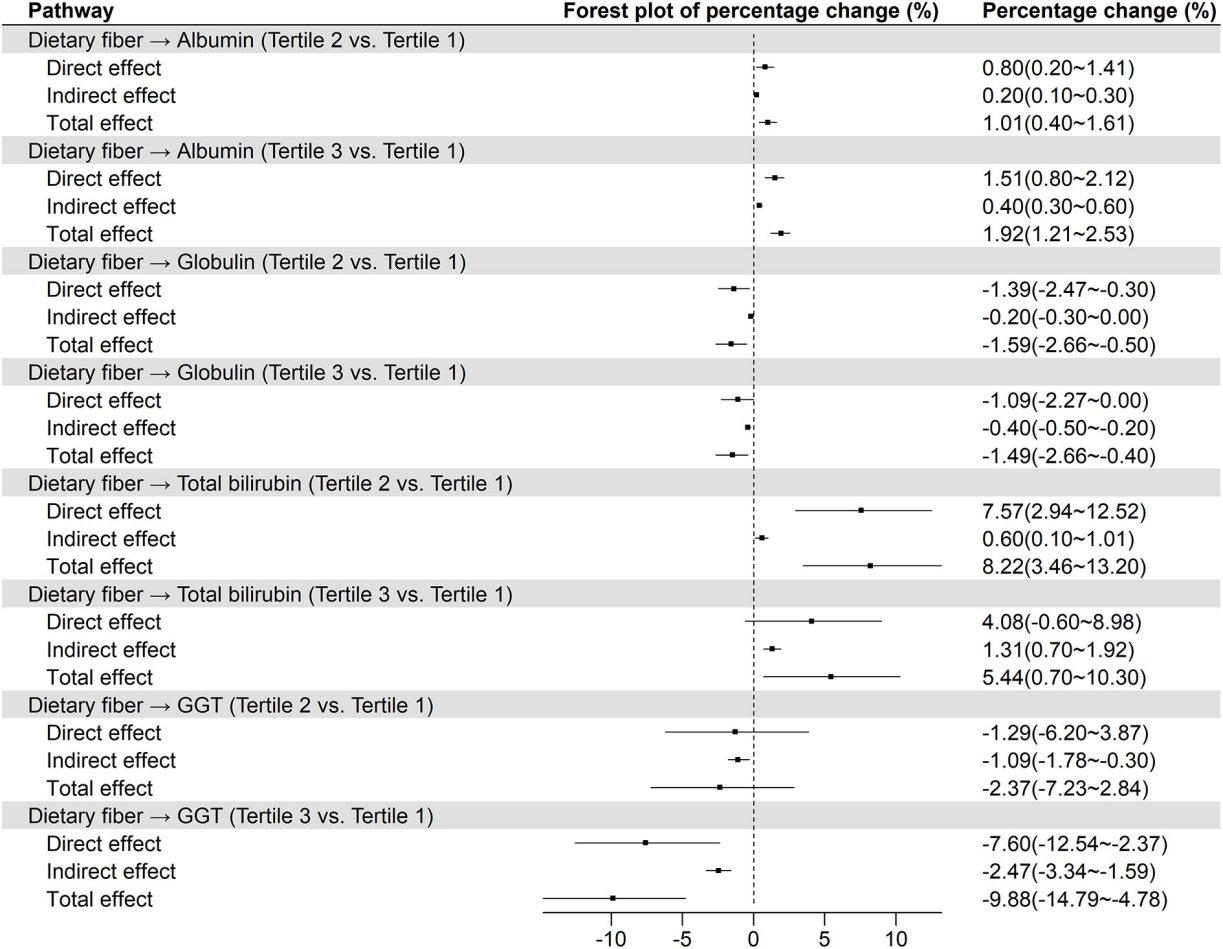
The relationship between dietary fiber and liver function parameters from mediation analysis with controlling obesity as a mediator^a^. GGT, Gamma-glutamyl transaminase. ^a^Model was adjusted for adjusted for age, sex, smoking, race/ethnicity, education, ratio of family income to poverty, physical activity, total energy, hypertension, and diabetes.

Furthermore, it was worth noting that liver function parameters varied across tertiles of dietary fiber consumption. After multivariable adjusted, the concentration of albumin and total bilirubin increased by 2.00% (95% CI: 1.33–2.67%) and 6.20% (1.43–11.20%) from the lowest tertile to the highest tertile of dietary fiber consumption, while concentration of globulin and GGT dropped by 1.53% (0.40–2.66%) and 9.80% (4.66–14.66%), respectively (Table 3). For the liver biochemical indicators, obesity mediated the association of dietary fiber with albumin, globulin, total bilirubin, and GGT (Figure 3). In comparing tertile 3 with tertile 1, 21.05%, 26.67%, 24.53%, and 24.04% of the total effects of dietary fiber on albumin, globulin, total bilirubin, and GGT were mediated by obesity, respectively. In addition, there were no statistically significant total effect of dietary fiber on total protein, ALT, and AST (Supplementary Figure 3).

**Table 3:** The associations of dietary fiber consumption with liver function parameters.

**Liver function parameters^a^**	**Dietary fiber consumption**			**Per 1-SD**	** $P_{trend}^{\text{d}}$ **

	**Tertile 1**	**Tertile 2**	**Tertile 3**		
**Albumin**					
Model 1^b^	0	0.62 (−0.02 to 1.25)	1.36 (0.72 to 2.00)	0.58 (0.33 to 0.84)	<0.001
Model 2^c^	0	1.08 (0.46 to 1.71)	2.00 (1.33 to 2.67)	0.84 (0.56 to 1.12)	<0.001
**Globulin**					
Model 1^b^	0	−1.67 (−2.71 to −0.62)	−0.68 (−1.73 to 0.38)	−0.25 (−0.69 to 0.18)	0.253
Model 2^c^	0	−1.61 (−2.68 to −0.53)	−1.53 (−2.66 to −0.40)	−0.74 (−1.21 to −0.26)	0.003
**Total protein**					
Model 1^b^	0	−0.41 (−0.88 to 0.06)	0.42 (−0.05 to 0.90)	0.20 (0.01 to 0.39)	0.044
Model 2^c^	0	−0.13 (−0.61 to 0.36)	0.42 (−0.10 to 0.94)	0.14 (−0.07 to 0.36)	0.198
**Total bilirubin**					
Model 1^b^	0	8.65 (4.22 to 13.27)	7.39 (3.02 to 11.95)	2.25 (0.52 to 4.00)	0.011
Model 2^c^	0	8.63 (3.99 to 13.47)	6.20 (1.43 to 11.20)	1.53 (−0.40 to 3.50)	0.121
**ALT**					
Model 1^b^	0	1.19 (−2.97 to 5.52)	2.38 (−1.82 to 6.76)	1.29 (−0.43 to 3.04)	0.143
Model 2^c^	0	0.82 (−3.38 to 5.20)	−0.08 (−4.46 to 4.50)	0.88 (−0.99 to 2.79)	0.360
**AST**					
Model 1^b^	0	1.42 (−1.34 to 4.27)	2.35 (−0.44 to 5.21)	1.05 (−0.08 to 2.20)	0.069
Model 2^c^	0	0.64 (−2.29 to 3.66)	0.80 (−2.29 to 3.98)	0.65 (−0.65 to 1.96)	0.331
**GGT**					
Model 1^b^	0	−2.55 (−7.45 to 2.61)	−7.84 (−12.47 to −2.97)	−3.83 (−5.84 to −1.78)	<0.001
Model 2^c^	0	−2.45 (−7.44 to 2.82)	−9.80 (−14.66 to −4.66)	−4.22 (−6.43 to −1.96)	<0.001

ALT, alanine aminotransferase; AST, aspartate aminotransferase; GGT, gamma-glutamyl transaminase.

a Liver function parameters were natural logarithm transformed. Percentage change (%) and 95% confidence intervals were calculated as (e^β^ – 1)^*^100% based on multivariable linear regression.

b Model 1 did not adjust for the covariates.

c Model 2 was adjusted for age, sex, smoking, race/ethnicity, education, ratio of family income to poverty, physical activity, total energy, hypertension, and diabetes.

^d^The trend test was performed by using z-score in the models.

## Discussion

When no mediator was considered, dietary fiber was inversely associated with NAFLD, and striking dose-response curves suggested that higher intake of dietary fiber could confer even greater benefit to protect against NAFLD. After obesity was controlled for the mediator, we found significant total and indirect association, yet there was no longer a significant direct association of dietary fiber with NAFLD. Our results indicated that obesity fully mediated the association of dietary fiber with NAFLD in this large cross-sectional study. Given the limited number of CSF, we did not observe a statistically significant association between dietary fiber and CSF.

We observed that obesity was strongly linked to NAFLD with an approximately fourfold increased risk. A previous study reported the prevalence of NAFLD up to 80% among obese adults, while 16% among adults with a normal BMI (26), and a meta-analysis reported a significant dose-response relationship between BMI and the risk of NAFLD (13). Even more alarming is that a similar association of obesity with NAFLD was observed among children and adolescents. A recent study of 408 US adolescents with obesity reported that the prevalence of NAFLD was up to 26.0% (27), and the findings from 1,900 adolescents in Italy showed that adolescents with high waist-to-height ratio had a significantly higher risk of NAFLD and elevated ALT (28). Although the underlying mechanisms of the associations between obesity and NAFLD are not yet fully understood, the adipose tissue (AT) expandability hypothesis can link obesity with the development of NAFLD (29). AT and liver share an evolutionary origin, when each individual adipose tissue reaches the limit of lipid storage, lipids are redirected toward liver and will begin to be deposited ectopically. One consequence of ectopic lipid accumulation is insulin resistance (IR), which is involved in the pathogenesis of NAFLD (30). Ectopic lipid accumulation in the hepatocytes leads to simple steatosis followed by the immune cells infiltrating in liver further contributing to a chronic intrahepatic inflammatory process and consequent fibrosis, a condition characterized as non-alcoholic steatohepatitis (NASH) (31).

Carbohydrates are the main source of energy. Recent studies have concerned about the health effects of carbohydrates quality rather than quantity (32). Thereinto, dietary fiber has long been thought to have health benefits. The evidence from epidemiology to clinical intervention trials suggested that a generous intake of dietary fiber could lower the risk of obesity and its related non-communicable diseases (33, 34). Observational data suggested an ~30% decrease in incidence of obesity when comparing the highest dietary fiber consumers with the lowest consumers (33). Furthermore, dietary fiber contributes to weight loss for obese population. A meta-analysis of clinical trials showed that higher intake of dietary fiber could help weight loss (34). Our finding of a reduction in risk of obesity among the highest dietary fiber consumers was consistent with previous epidemiological studies. This association is supported by some biological evidence. Fiber-rich food usually requires more chewing times, leading to increased satiety and reduced food intake (35). which may be beneficial in controlling body weight. On the other hand, dietary fiber lowers the risk of obesity through microbiome-related mechanisms. Dietary fiber is fermented by microbiota within gastrointestinal tract and converted to short chain fatty acids (SCFAs), predominantly acetate, propionate, and butyrate. SCFAs can activate G-protein-coupled receptors (*GPR41* and *GPR43*), ultimately suppressing appetite to prevent weight gain. Likewise, butyrate has been shown to be protective against obesity through increasing energy expenditure (34).

A previous study suggested that BMI might be a mediator in the pathway between dietary factor and NAFLD (14), however, the indirect effect mediated through obesity was not considered in the analysis. According to the present study, the mediation analysis showed that most of the association appeared to have been an indirect effect mediated through obesity. Although we did not observe a statistically significant direct effect of dietary fiber on NAFLD, several evidences supported that dietary fiber could also influence the onset of NAFLD through other biological mechanisms. First, the gut and liver bidirectionally communicate through the biliary tract and portal vein, termed gut-liver axis. A well-known effect of dietary fiber is to regulate gut microbiota, a component of the intestinal barrier. When the gut barrier is compromised, liver is the first extraintestinal organ to be exposed to bacteria and bacterial products, causing inflammation and hepatic injury (36). Second, SCFAs, produced by fermentation of dietary fiber by intestinal microbiota, protect against NAFLD primarily through modulation of inflammation. SCFAs binding to *GPR43* recruit immune cells and regulate inflammatory responses. The progression of NAFLD is often hallmarked by immune cell infiltration. SCFAs reduce liver inflammatory responses by inhibiting the activity of histone acetyltransferases (37). In addition to reducing hepatic inflammatory responses, SCFAs can regulate hepatic lipid metabolism. SCFAs promoted energy expenditure and lipid oxidation through an adenosine monophosphate-activated protein kinase (AMPK) dependent mechanism, reducing the risk of NAFLD (37). Conversely, SCFAs also appear to promote NAFLD. Increased SCFAs flow into the liver through the portal vein, contributing to triglyceride accumulation and gluconeogenesis in the liver (38).

Liver disease develops silently with no signs or symptoms until the late stages. Liver function tests may contribute to the early detection of diseases. A cross-sectional study with 265 healthy adults reported that individuals in the highest quartile of vegetable intake were less likely to have elevated ALT (39), and animal experiment also demonstrated that supplements of insoluble and soluble fibers lowered serum levels of ALT and AST, while increasing serum level of albumin and total protein (40). In current study, we also evaluated the association between dietary fiber and NAFLD at the biochemical level, and observed a similar result that dietary fiber could ameliorate liver function. Elevated serum albumin and total bilirubin were observed among participants with high intake of dietary fiber. Albumin is exclusively synthesized by the liver, and low albumin levels may be a marker of advanced diseases in chronic liver inflammation or cirrhosis (41). Bilirubin is the end product of the breakdown of red blood cells. Accumulating evidence indicates that higher bilirubin levels are inversely associated with NAFLD (42, 43), although the underlying mechanisms are not well elucidated. On the other hand, decline of serum globulin, and GGT was observed among adults with high intake of dietary fiber. Globulin is a group of proteins synthesized mainly in liver by immune system, increase in globulin may indicate inflammatory diseases (44). GGT is released from damaged liver cells into the blood after hepatocellular injury or death (45, 46), those are considered to serve as reliable non-invasive biomarkers of liver injury.

By combining the findings, dietary fiber intake was associated with lower odds of NAFLD through obesity. Consumption of whole grains and vegetables are important contributors to dietary fiber intake. A case-control study enrolled 940 NAFLD and 940 age- and sex-matched controls from Chinese adults, reported that risk of NAFLD gradually decreased across increasing tertiles of plasma 3-(3,5-dihydroxy phenyl)-1-propanoic acid, a biomarker of whole-grain wheat and rye intake (47). In a randomized controlled clinical trial, 112 patients with NAFLD were randomly assigned to obtain at least half of cereal each day from whole-grain foods or usual cereals for 12 weeks. A substantial decrease in grades of fatty liver, ALT, AST, and GGT was observed after 12 weeks on the whole-grain foods (48). Furthermore, a cross-sectional study of 18,345 US adults suggested that plant-based diet index (PDI), *a priori* dietary pattern, was negatively associated with ALT, AST, and FLI. Moreover, the highest tertile of PDI was found to reduce a fifth odd of NAFLD (6).

A major strength of the present study was that it has related dietary fiber to NAFLD through controlling obesity. Several limitations should be addressed. First, dietary information was surveyed using two 24-h recalls, it was difficult to capture the long-term dietary intake of dietary fiber. Second, limited data were available regarding specific sources of dietary fiber, we did not estimate the associations of fiber according to diverse food sources with NAFLD risk. Third, we did not observe the obvious threshold effect, although there was a negative association between dietary fiber intake and NAFLD as well as CSF. Moreover, the *Dietary Guidelines for Americans, 2020–2025* recommend dietary fiber intake of 34 g/1,000 kcal per day for American adult men, and 28 g/1,000 kcal per day for American adult women, respectively (49). This was consistent with the findings of our study that the risk of both NAFLD and CSF was gradually decreased when the fiber intake was within 34 g/1,000 kcal per day. Additionally, the cross-sectional design in the current study did not allow the determination of causality. Through this study alone, it was difficult to provide an exact cut-off value, and large-scale prospective studies or clinical intervention trials in the future were warranted to provide more accurate guidance on the proportion of dietary fiber intake.

Taken together, the present study demonstrated that increased intake of dietary fiber was associated with lower odds of NAFLD. The findings contribute to the growing body of evidence that increasing dietary fiber intake from plant foods or supplements could confer a greater benefit to protect against NAFLD and improve liver function. Translating these findings regarding dietary fiber into dietary advice might be an attractive strategy for NAFLD prevention.

## Data availability statement

The datasets presented in this study can be found in online repositories. The names of the repository/repositories and accession number(s) can be found below: https://wwwn.cdc.gov/nchs/nhanes/continuousnhanes/default.aspx?BeginYear=2017.

## Ethics statement

The studies involving human participants were reviewed and approved by NCHS Research Ethics Review Board. The patients/participants provided their written informed consent to participate in this study.

## Author contributions

Full access to all the data in the study and take responsibility for the integrity of the data and the accuracy of the data analysis and drafting of the manuscript: YZhu and HY. Study concept and design: WY and YZhu. Statistical analysis: YZhu. Obtained funding, administrative, technical, or material support, and study supervision: WY. Acquisition, analysis, or interpretation of data, and critical revision of the manuscript for important intellectual content: all authors. All authors contributed to the article and approved the submitted version.
