# Supplementary material for: Dietary fiber intake and non-alcoholic fatty liver disease: The mediating role of obesity
Source: Front Public Health. 2023 Jan 6;10:1038435. doi: 10.3389/fpubh.2022.1038435 (PMC9853063; doi:10.3389/fpubh.2022.1038435)
Supplement: Supplementary file 1 [file Data_Sheet_1.DOCX]

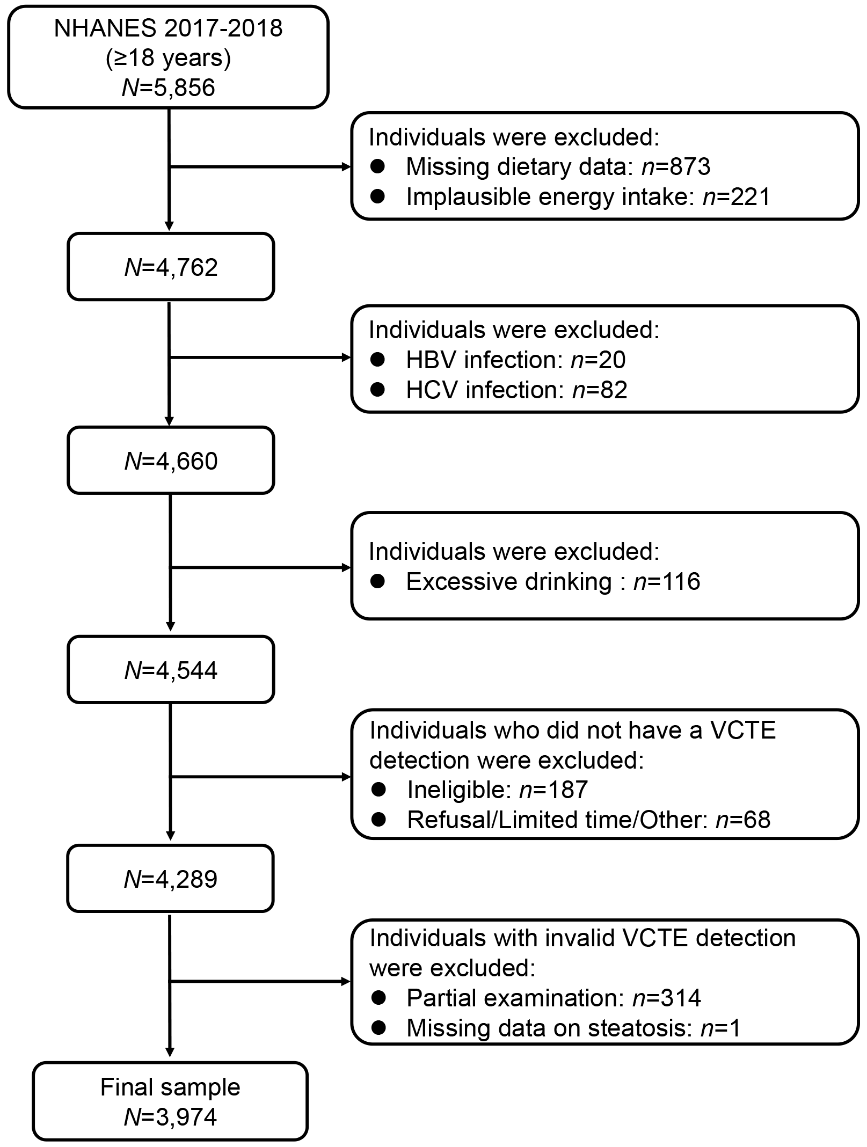


**Fig****ure S1.** Flow chart of the study participants from the 2017-2018 cycle of NHANES ^a^.

^a^ Abbreviations: HBV: Hepatitis B Virus; HCV: Hepatitis C Virus; NHANES: U.S. National Health and Nutrition Examination Survey; VCTE: Vibration-controlled transient elastography.


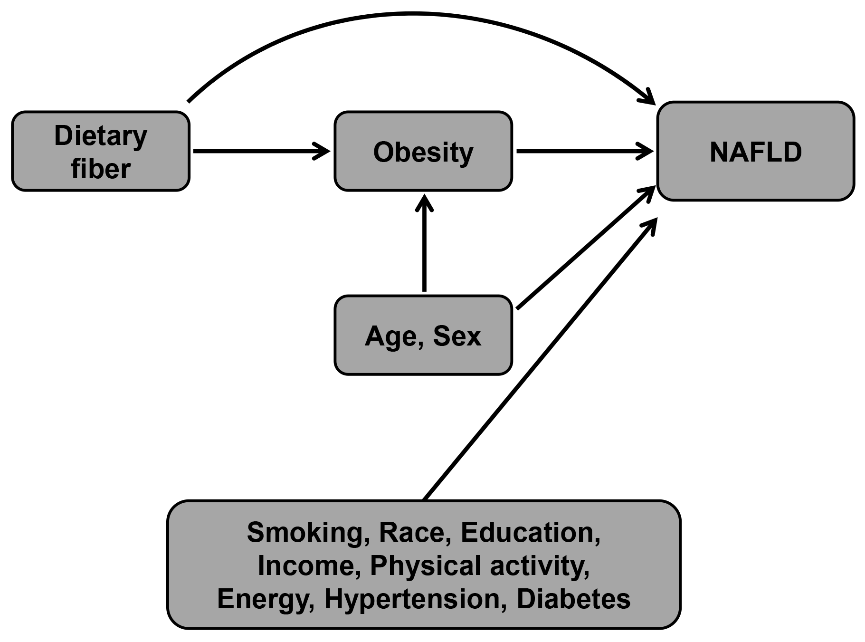


**Figure S2.** Pathway of the mediation process for the association of dietary fiber intake with NAFLD phenotypes.


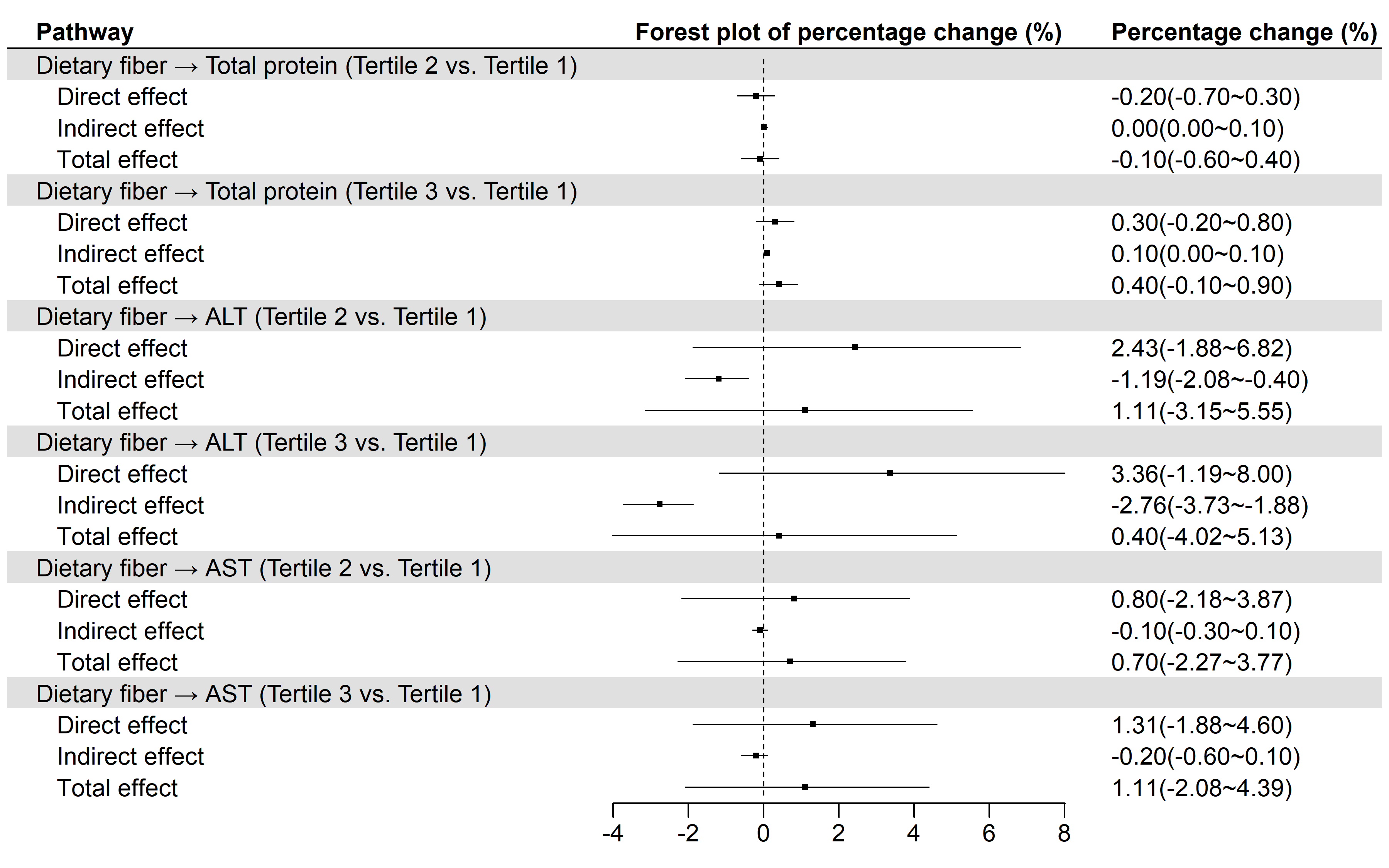


**Figure S3.** The relationship between dietary fiber and liver function parameters from mediation analysis with controlling obesity as a mediator ^a^.

Abbreviations: ALT, Alanine aminotransferase; AST, Aspartate aminotransferase.

^a^ Model was adjusted for adjusted for age, sex, smoking, race/ethnicity, education, ratio of family income to poverty, physical activity, total energy, hypertension, and diabetes.
